# Association between initial opioid use and response to a brief interdisciplinary treatment program in fibromyalgia

**DOI:** 10.1097/MD.0000000000013913

**Published:** 2019-01-04

**Authors:** Jong-moon Hwang, Byung-joo Lee, Terry H. Oh, Donghwi Park, Chul-hyun Kim

**Affiliations:** a Department of Rehabilitation Medicine, Kyungpook National University Hospital; b Department of Rehabilitation Medicine, School of Medicine, Kyungpook National University, Daegu, Korea; c Department of Physical Medicine and Rehabilitation, Mayo Clinic, Rochester, MN; d Department of Rehabilitation Medicine, Daegu Fatima Hospital, Daegu, South Korea.

**Keywords:** fibromyalgia, Fibromyalgia Impact Questionnaire, fibromyalgia treatment program, opioid, quality of life, Short Form-36 Health Status Questionnaire, symptom

## Abstract

**Background:**

To evaluate the association between opioid use and treatment outcome (symptom severity, quality of life [QOL]) after a brief interdisciplinary fibromyalgia treatment program (FTP).

**Method:**

Subjects (n = 971) with fibromyalgia participated in the FTP. They filled out the Fibromyalgia Impact Questionnaire (FIQ) and the Short Form-36 Health Status Questionnaire (SF-36) at baseline and 6 to 12 months after the FTP. Post-treatment changes in FIQ and SF-36 scores were compared after stratifying the participants into opioid user and non-opioid user groups.

**Results:**

A total of 236 patients (24.3%) were opioid users. Compared with non-opioid users, the opioid users had worse symptom severity measured using FIQ total score (*p* < .001) and all subscales at baseline and post treatment, as well as worse QOL measured using all SF-36 subscales and physical and mental components. Comparison of least-square means of mean change of QOL between opioid users and non-opioid users after analysis of covariance adjusted patient characteristics and baseline scores showed that the FIQ subscale scores of physical impairment (*p* < .05), job ability (*p* < .05), and fatigue (*p* < .05) were significantly less improved in the opioid users compared with the non-opioid users. Moreover, the SF-36 subscale score of general health perception (*p* < .05) was significantly less improved in the opioid users compared with non-opioid users. However, post-treatment changes in mean scores for QOL subscale generally did not significantly differ in both groups.

**Conclusions:**

Opioid use did not affect response to the FTP, as measured using the FIQ total score or SF-36 physical and mental component summary scores. Furthermore, the opioid user group showed less improvement in the FIQ subscale scores of physical impairment, job ability, and fatigue and in the SF-36 subscale scores of general health perception.

## 1 Introduction

Fibromyalgia is a condition characterized by chronic, widespread, musculoskeletal pain with decreased pain threshold to pressure or other stimuli.^[1,2]^ It is often associated with various physical and psychological symptoms. These symptoms include headache, bowel and bladder symptoms, fatigue, sleep disturbance, cognitive dysfunction, headache, and depression.^[3]^ Extensive research has revealed that central sensitization is the main pathophysiologic feature of fibromyalgia. Central sensitization is characterized by augmented processing of pain stimulation that is managed partially through peripheral mechanisms. Therefore, evidence-based pharmacological therapy for fibromyalgia focuses on alterations in descending inhibitory pathways and other pain-processing pathways. Despite little evidence supporting the long-term effect of opioid treatment for fibromyalgia, opioid therapy in this population ranges from 24% to 30%.^[4–7]^

Currently, the use of opioids for the treatment of chronic pain is widespread.^[8]^ However, further knowledge on the effect of opioids in fibromyalgia is crucial because previous studies suggested that patients who use opioids have worse outcomes in pain-related physical and emotional function.^[9]^ Consequently, this study aimed to investigate the effect of opioid use on outcome of fibromyalgia treatment, specifically interdisciplinary fibromyalgia treatment program (FTP).

## 2 Materials and methods

### 2.1 Participants

The study population was part of the earlier study report by our group, regarding 6 to 12-month treatment outcome after our FTP.^[10]^ Each patient had a confirmed diagnosis of fibromyalgia according to the American College of Rheumatology 1990 criteria for fibromyalgia classification.^[1]^ Patients underwent the FTP from May 1, 2001, to April 30, 2004. All patients agreed to participate in the study. The study was approved by the Mayo Clinic Institutional Review Board, and informed consent was obtained from each patient before participating in the study. The patients were not self-referred nor externally referred. This study population consisted of 917 patients who agreed to complete the Fibromyalgia Impact Questionnaire (FIQ) and the Short Form-36 Health Status Questionnaire (SF-36) at baseline and 6 to 12 months after participating in the FTP.

### 2.2 Opioid user and nonuser grouping

At the first visit for the FTP, a patient was evaluated by a registered nurse (RN) who would use “shorthand” computer software to collect standardized information while the evaluation was being conducted. This comprehensive assessment included current symptoms, impact of pain, medical history, and other symptoms on physical and emotional functioning. The nurse also performed a tender point examination.

All patients participating in the FTP during this time period were internally referred within the institution, which used the same computer operating software. A complete list of medication was organized by former providers before referral to the FTP. The RN reconducted a medication recognition process, confirming with the patient and updating any details related to medication use, to ensure an accurate medication list. Meanwhile, the RN asked in a nonjudgmental, matter-of-fact manner if the patient used any nonprescribed material to assist them in symptom management. Substances, such as cocaine, heroin, marijuana, and opioids, were provided as examples that were prescribed for another person. Finally, if the patient had any records from the local physician, the RN would review and remark them as prior medications in the patients’ current medical record. Opioid user was defined as patients who were active users at the time of the FTP evaluation. Participants who used opioids before but were not using at the time of the evaluation were included in the nonopioid user group. Patients who had stopped using opioids the day before the evaluation were also considered in the nonopioid group. Moreover, patients using tramadol were included in the nonopioid user group. Despite the weak μ-receptor agonist property of tramadol, several studies have shown some positive effects in fibromyalgia management^[11,12]^ and hence has been included in treatment recommendations and guidelines for fibromyalgia.^[13–15]^ The pain control effects of tramadol in fibromyalgia are speculated to be mainly from inhibition of norepinephrine and serotonin and reuptake.^[16]^

### 2.3 Outcome measure

The FIQ and SF-36 were used to measure treatment outcome. The FIQ, developed in 1991, is a validated, practical tool used to assess the health status of patients with fibromyalgia.^[17]^ This self-administered questionnaire evaluates multiple components of symptoms and functional impairment related to fibromyalgia through 20 questions. The included 10 items are as follows: physical functioning, fatigue, symptoms of pain, morning tiredness, job difficulty, stiffness, depression, anxiety, days of work missed, and overall well-being in the previous week. Result score ranges from 0 to 100, with 100 demonstrating maximum impairment. The FIQ was performed according to the directions specified by Bennett.^[18]^

The SF-36 assesses health-related quality of life (QOL). It was developed as a tool for evaluating patient outcomes in a busy clinical setting.^[19]^ The self-administered questionnaire measures 8 health concepts, namely, role limitation due to physical health, physical functioning, bodily pain, general health perception, social role functioning, vitality score, role limitations due to emotional health and mental health, and physical and mental composite scores. The SF-36 score ranges from 0 to 100, with higher score indicating better health status.^[20]^

Patients completed the FIQ and SF-36 at the initial visit. The same questionnaires were mailed 6 and 12 months after FTP completion. If no response was received within 1 month after mailing, the questionnaires were remailed. We did not communicate with the patients through phone or otherwise persuade them to complete the follow-up questionnaires. Both returned questionnaires were used for our analysis.

### 2.4 Statistical analysis

R language version 3.3.3 (R Foundation for Statistical Computing, Vienna, Austria), T&F program ver. 2.5 (YooJin BioSoft, Korea), and IBM SPSS Statistics for Windows version 22 (IBM Corp, Armonk, NY) were used for all statistical analyses. Data were expressed as mean ± SD for continuous variables. When variables were normally distributed, mean difference test between 2 sample groups was performed using Student *t* test. For non-normally distributed variables, Mann–Whitney *U* test was used. For categorical variables, data were expressed as sample number and percentage, N (%). Chi-squared test or Fisher exact test was performed to compare proportions of sample number as appropriate.

When variables were normally distributed, paired sample *t* test was used to test if the mean difference between paired sets of observations was zero. For non-normally distributed variables, Wilcoxon signed-rank test was performed. The paired sets are FIQ and SF-36 scores measured at baseline and post-treatment.

Analysis of covariance (ANCOVA) was performed to evaluate whether population means of response were equal across sublevels of group variable (opioid users vs nonusers) while statistically controlling for the effects of confounding covariates such as age, sex, body mass index (BMI), tobacco use, alcohol use, employment status, benzodiazepine use, and FIQ or SF-36 scores measured at baseline. Response consisted of FIQ and SF-36 scores measured at baseline and post-treatment. Mean change of FIQ and SF-36 scores were also used as response.

## 3 Results

### 3.1 Patients

Our study population consisted of 971 patients (917 women and 54 men), with a mean (SD) age of 49 (12.92) years and a mean (SD) duration of symptoms of 132.45 (134.52) months in the nonopioid users. In the opioid users, a mean (SD) age was 47.55 (12.33) years and mean duration of symptoms was 123.21 (135.55) months. Among these patients, 236 (24.3%) were opioid users and 735 (75.7%) were nonopioid users. The patient characteristics of opioid and nonopioid users are outlined in Table 1.

**Table 1:**
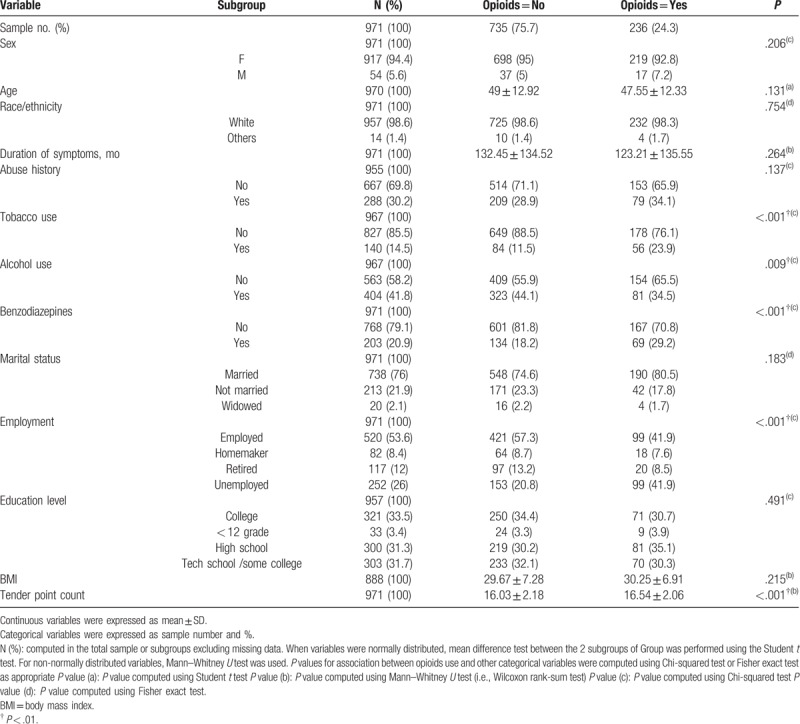
Patient characteristics of opioids users and nonopioid users.

### 3.2 QOL baseline scores and post-treatment changes by opioid use

The opioid users showed a higher severity of symptoms measured using the FIQ total score and all subgroups at baseline and post-treatment (Table 2) than the nonopioid users. Similarly, the opioid users had worse QOL when measured in all SF-36 subscales and SF-36 physical and mental components (Table 3). However, after the FTP, both groups achieved improvement in the FIQ total score and all FIQ subscales except depression. Depression (FIQ subscales) was exacerbated in the group that did not use opioids for treatment (Table 2). The SF-36 subscales were also improved except for general health perceptions. In the opioid user group, the general health perceptions subscale was worse (Table 3).

**Table 2:**
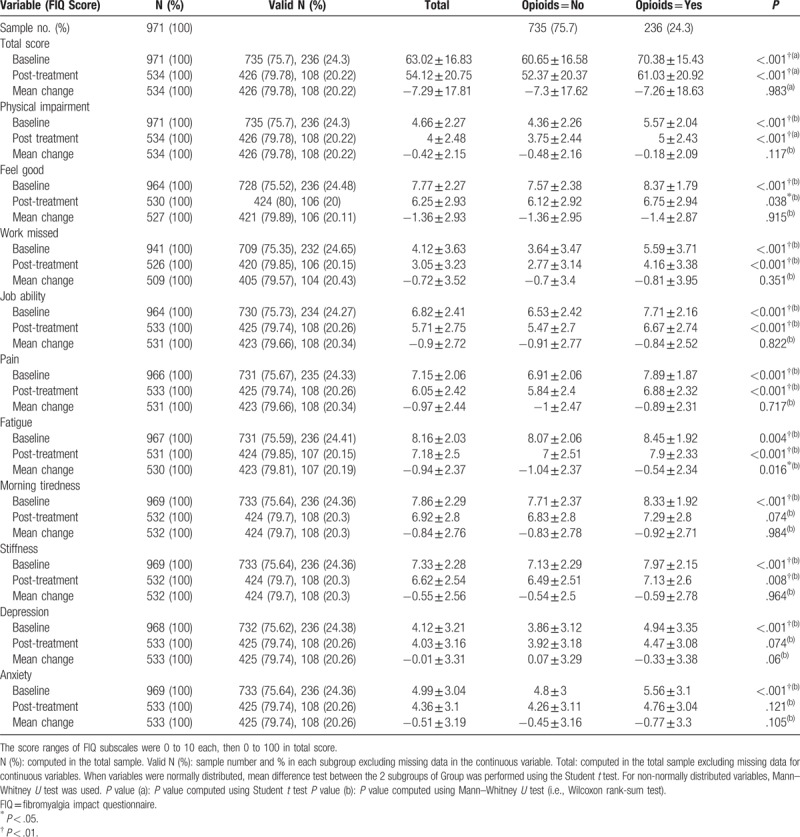
FIQ baseline scores and post-treatment changes by opioid use.

**Table 3:**
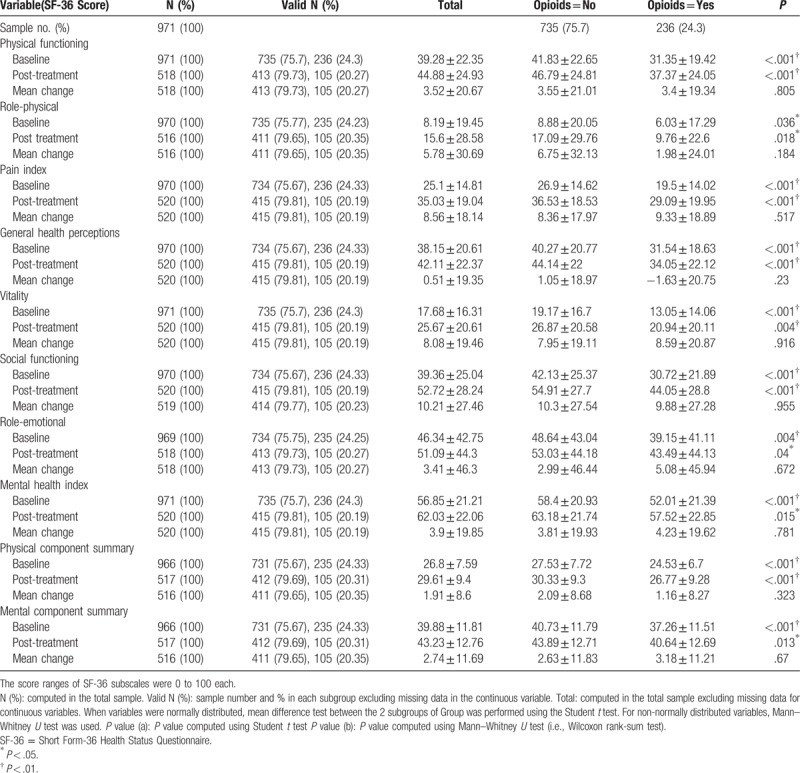
SF-36 baseline scores and post-treatment changes by opioid use.

#### 3.2.1 Comparison of least-square means of mean change of QOL between opioid users and nonopioid users

**Figure 1:**
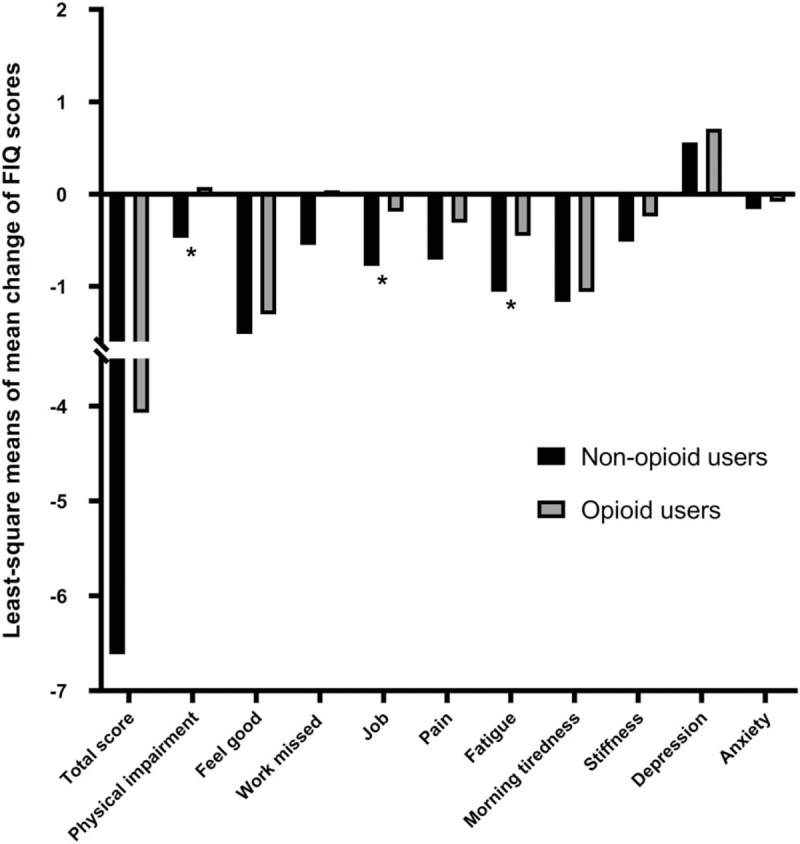
Comparison of least-square means of mean change of FIQ (Fibromyalgia Impact Questionnaire) subscale scores between opioid users and nonopioid users. ^∗^*P* < .05.

**Figure 2:**
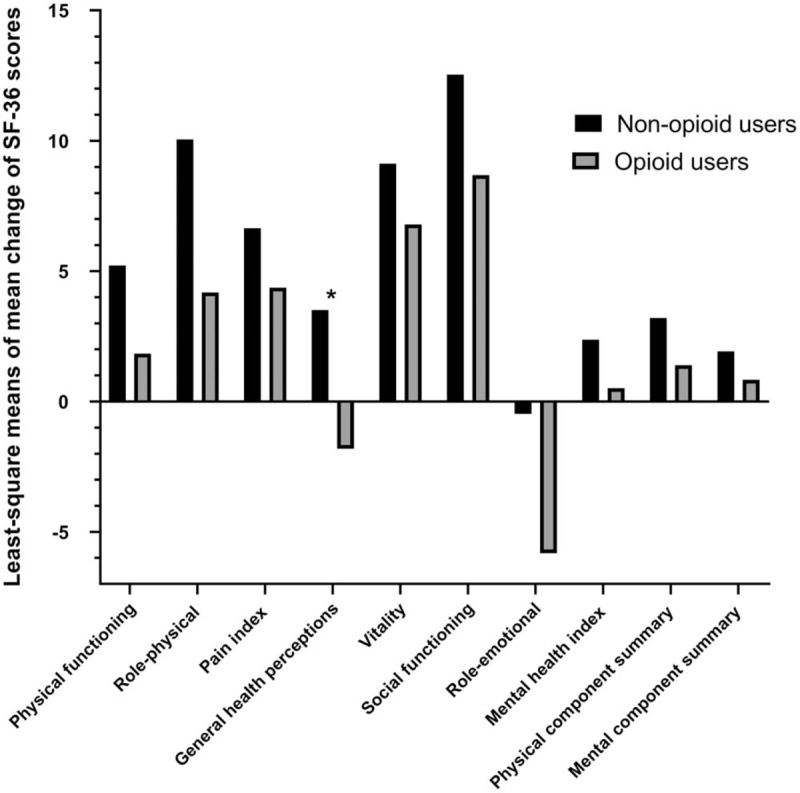
Comparison of least-square means of mean change of SF-36 (Short Form-36 Health Status Questionnaire) subscale and summary scores between opioid users and nonopioid users. ^∗^*P* < .05.

After ANCOVA was used to adjust for patient characteristics (tobacco use, alcohol use, employment, benzodiazepines, age, sex, BMI) and baseline scores, the FIQ subscale scores of physical impairment (*P* = .017), job ability (*P* = .038), and fatigue (*P* = .04) were significantly less improved in the opioid users compared with the nonopioid users. The SF-36 subscale score of general health perception (*P* = .013) was also significantly less improved in the opioid users compared with the nonopioid users. However, post-treatment changes in mean scores for QOL subscale generally did not significantly differ in both groups. The changes in FIQ and SF-36 scores in both groups are summarized in Tables 4 and 5, respectively, and are plotted in Figs. 1 and 2.

**Table 4:**
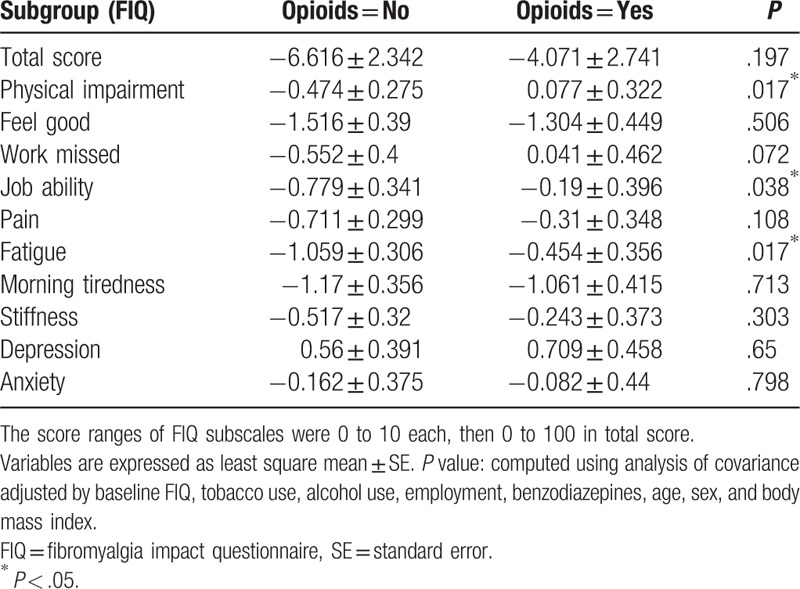
Comparison of least-square means of mean change of fibromyalgia impact questionnaire scores between opioid users and nonopioid users.

**Table 5:**
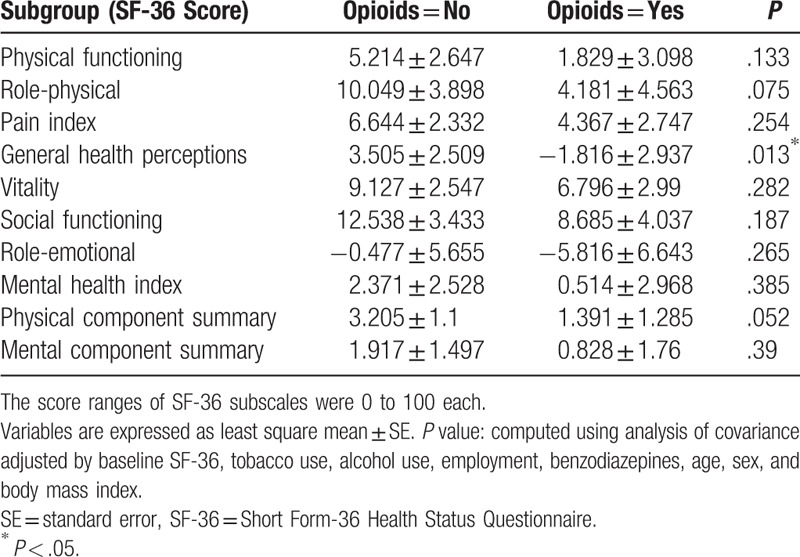
Comparison of least-square means of mean change of Short Form-36 Health Status Questionnaire scores between opioid users and nonopioid users.

## 4 Discussion

The important finding of this study was that the use of opioids in fibromyalgia does not improve the outcome of the treatment program. Improvements were noted for the total measures of function, pain, and mood, regardless of opioid status. However, the opioid user group constantly scored higher than the nonopioid user group in all measures of symptom severity, with significance prominent for higher pain scores and worse functional impairment. The results suggest that despite improvements in pain, function, and psychological variables during a multidisciplinary pain clinic care, these measures were irrespective of opioid use. The use of opioids was not associated with improvement of disease status beyond what was observed from previous standard multidisciplinary treatment setting. Furthermore, work status of opioid users was less favorable with more disability payments noted and unemployment for the opioid user group. These findings raise questions regarding the rational use of opioids in patients with fibromyalgia.

Pain response is presently used as a significant outcome measurement for treatment assessment in clinical trials of fibromyalgia.^[21]^ However, the efficacy of any treatment method in fibromyalgia should reflect both improvement in the target symptoms and functional status. Opioid therapy has been established in the management of chronic pain. Our findings of 20% prevalence of opioid use in patients with fibromyalgia are consistent with studies of opioid use in patients with chronic nonmalignant painful conditions.^[22]^ In fact, treatment with any analgesic other than nonsteroidal anti-inflammatory drugs was reported by more than half of patients with fibromyalgia in a survey conducted in the United States.^[23]^ Patients with fibromyalgia evidently consider opioids as the best symptom reliever.^[24]^ According to an Internet survey of people with fibromyalgia, opioids were reported to be the most helpful treatment of pain.^[24]^

While the use of opioids in the management of fibromyalgia pain remains controversial, their known analgesic properties should encourage further investigation in fibromyalgia treatment. Only tramadol has been formally studied in fibromyalgia management to date, with a positive effect on pain and QOL.^[11,25]^ However, opioid use is not supported by any guidelines for the management of fibromyalgia.^[13,26,27]^

A number of factors need to be considered with regard to the worse outcome in patients on opioid treatment. Opioid users possibly had more severe disease course or had more comorbidities resulting in continuous opioid use as has been previously mentioned.^[28]^ Whereas demographic variables did not differ between patients stratified according to opioid use, the opioid group evidently showed more functional impairment, pain, and mood disorder at baseline compared with the nonopioid group. This may suggest that the opioid users were more symptomatic from the beginning.

Despite the expectation of showing a better clinical outcome related to opioid use, we have observed that the opioid group, even when longitudinally followed by a multidisciplinary team, was more functionally impaired and symptomatic. This raised the question whether opioids per se may have contributed to our results due to the effects of higher pain associated with central sensitization.^[29]^ This suggestion is conceivable, as symptoms of fibromyalgia and adverse effects of opioid use are markedly similar, such as deteriorated physical and mental performance, fatigue, and even worse pain. In accordance with this observation, our result suggests that opioid use is somewhat common but is associated with worse fibromyalgia-associated symptoms and poorer QOL measures. A recent 1-year observational study on patients with fibromyalgia showed worse outcomes in opioid users in functioning, depression, daily activities, and insomnia compared with nonopioid users and tramadol users.^[9]^ However, the mechanisms mediating the adverse effects of opioids in this particular patient population are poorly understood. In a previous study, patients with fibromyalgia showed a decreased central μ opioid receptor availability.^[30]^ This alteration in the endogenous opioid system has been suggested to negatively influence the efficacy of opioids^[15]^ and worsen the sensitization of pain-associated neural pathways.^[31,32]^ In another report, patients with fibromyalgia had an increased level of endogenous enkephalins in cerebrospinal fluid.^[33]^

Despite vigorous studies concerning the mechanism of opioids in fibromyalgia, without definite evidence which supports the effect of opioids on patients with fibromyalgia, their use should be considered with caution.

The present study had several limitations. First, the patients in this study cohort were treated at a tertiary medical center. Therefore, they may not be representative of all patients with fibromyalgia. Second, most patients who participated in this study already had symptoms for more than 10 years. Therefore, we could not determine the severity of symptoms neither at onset of disease nor in the absence of treatments. Third, data collection had no accurate assessment of opioid dose or duration of opioid use. Fourth, we did not use the updated American College of Rheumatology criteria for fibromyalgia, as our study participants were assessed from 2001 to 2004. However, the previous version of the criteria has more strict standards than the updated version. Therefore, no controversy exists with regard to patient recruitment.

## 5 Conclusion

Both opioid and nonopioid groups showed improvement in QOL and functional ability based on FIQ and SF-36 scores. However, opioid use did not affect response to the FTP, as measured using the FIQ total score or SF-36 physical and mental component summary scores. Furthermore, the opioid user group showed less improvement in the FIQ subscale scores of physical impairment, job ability, fatigue, and SF-36 subscale scores of general health perception. While opioids remain a treatment choice for fibromyalgia, physicians should consider these study results and be more attentive with regard to the need for opioid prescription.

## Author contributions

**Conceptualization:** Jong-moon Hwang, Terry H. Oh, Donghwi Park, Chul-hyun Kim.

**Data curation:** Jong-moon Hwang, Terry H. Oh.

**Supervision:** Donghwi Park.

**Writing – original draft:** Jong-moon Hwang, Chul-hyun Kim.

**Writing – review & editing:** Jong-moon Hwang, Byung-joo Lee, Chul-hyun Kim.
